# Mild Acquired Factor XIII Deficiency and Clinical Relevance at the ICU—A Retrospective Analysis

**DOI:** 10.1177/10760296211024741

**Published:** 2021-07-21

**Authors:** Felix Carl Fabian Schmitt, Maik von der Forst, Wolfgang Miesbach, Sebastian Casu, Markus Alexander Weigand, Sonja Alesci

**Affiliations:** 1 Department of Anaesthesiology, Heidelberg University Hospital, Heidelberg, Germany; 2 Haemostaseology, Department of Internal Medicine II, Institute of Transfusion Medicine, University Hospital, Goethe University Frankfurt am Main, Frankfurt, Germany; 3 Department of Emergency Medicine, Asklepios Klinik Wandsbek, Hamburg, Germany; 4 Institute of IMD Blood Coagulation Centre, Frankfurt/Bad Homburg, Germany

**Keywords:** hemorrhage, factor XIII, critical care, blood transfusion, blood coagulation

## Abstract

Acquired FXIII deficiency is a relevant complication in the perioperative setting; however, we still have little evidence about the incidence and management of this rarely isolated coagulopathy. This study aims to help find the right value for the substitution of patients with an acquired mild FXIII deficiency. In this retrospective single-center cohort study, we enrolled critically ill patients with mild acquired FXIII deficiency (>5% and ≤70%) and compared clinical and laboratory parameters, as well as pro-coagulatory treatments. The results of the present analysis of 104 patients support the clinical relevance of FXIII activity out of the normal range. Patients with lower FXIII levels, beginning at <60%, had lower minimum and maximum hemoglobin values, corresponding to the finding that patients with a minimum FXIII activity of <50% needed significantly more packed red blood cells. FXIII activity correlated significantly with general coagulation markers such as prothrombin time, activated partial thromboplastin time, and fibrinogen. Nevertheless, comparing the groups with a cut-off of 50%, the amount of fresh frozen plasma, thrombocytes, PPSB, AT-III, and fibrinogen given did not differ. These results indicate that a mild FXIII deficiency occurring at any point of intensive care unit stay is also probably relevant for the total need of packed red blood cells, independent of pro-coagulatory management. In alignment with the ESAIC guidelines, the measurement of FXIII in critically ill patients with the risk of bleeding and early management, with the substitution of FXIII at levels <50%-60%, could be suggested.

## Introduction

Factor XIII, also known as fibrin stabilizing factor, is the latest clotting factor to be discovered, influencing processes like wound healing and inflammation, among others.^1–4^ Factor XIII is synthesized in the liver, platelets, and in macrophages/monocytes.^5–7^ A wide range of FXIII activity levels are described in the healthy population: from 53.2% to 221.3% and from 51% to 152% in 2 different studies.^8,9^ The effects on hemostasis are an enhanced clot stability, correlating with FXIII activity, and the crosslinking of alpha-2 plasmin inhibitor to fibrin, though early fibrinolysis is impaired.^10,11^ Congenital FXIII deficiency, partly with first symptoms at birth (e.g. prolonged or delayed umbilical bleeding and spontaneous cranial hemorrhage)^8,12^ and acquired FXIII deficiency, for example caused by major surgery, massive hemorrhage, sepsis, or acute liver failure are described.^13,14^ The delayed onset of these bleeding episodes, from 12-36 hours, is pathognomonic of low FXIII activity.^15^ While congenital deficiency FXIII levels below 10% are described as a cut-off for substitution, a high-risk for spontaneous bleeding is defined at levels below 4%. It is still unclear which is the right cut-off value for patients with an acquired deficiency.^8,13,16^ Previous publications have already shown that decreased FXIII levels might be an indicator for re-exploration after cardio-thoracic surgery and anastomotic insufficiency.^17,18^ Therefore, FXIII activity levels of 50%-60% have been suggested in the past to prevent major perioperative complications.^19–22^

In this context, it is important to classify the incidence and the need for treatment, especially for mild acquired FXIII deficiency, for a more evidence-based use of the different therapeutic options. In the past FXIII deficiency therapy consisted of the application of fresh frozen plasma (FFP) or cryoprecipitate. Today a plasma derived FXIII concentrate (Fibrogammin® P/Corifact™) and a recombinant FXIII product (containing only the A subunit) are available for targeted substitution of Factor XIII.^23,24^ In previous analyses regarding safety and efficacy, the use of the plasma derived product, as well as the application of recombinant FXIII, revealed a favorable safety profile.^25,26^

In this retrospective single-center study, we enrolled patients with mild acquired FXIII deficiency (>5% and ≤70%) in an intensive care unit (ICU) and compared the clinical and laboratory parameters, as well as the pro-coagulatory treatments, of the different FXIII activity levels. The intention was to gain an idea of the level at which the acquired FXIII deficiency causes clinically relevant problems, and to find out if there are possible implications for the management of perioperative mild factor XIII deficiency.

## Methods

The underlying study was planned as a retrospective cohort analysis. The study was conducted at 2 ICUs of the University Hospital Frankfurt. The period of recruitment was from October 2011 to July 2013. We screened for patients with mild Factor XIII deficiency in the ICU. The term “mild” was defined according to previous publications, as a FXIII activity under the normal range (<70%), but over 5%. In distinction to “moderate” (1%-4%) and “severe” (<1%) deficiency under normal conditions patients within this range do not show spontaneous bleeding or other specific symptoms of FXIII deficiency.^27,28^ The photometric determination of the activity of factor XIII in plasma samples was carried out with Berichrom® FXIII from Siemens (Marburg, Germany). In most cases, FXIII was analyzed routinely once a week.

Inclusion criteria were age >18 years and FXIII values under the normal range (≤70%). Exclusion criteria were bleeding disorders other than FXIII and not fulfilling the inclusion criteria. We enrolled 104 patients in this analysis. Demographic data and laboratory parameters, as well as the number of blood products and pro-coagulatory substances given during the period of intensive care, were evaluated. The follow-up ended with Dismission from intensive care unit.

Statistical data were collected with an electronic database system (Microsoft Excel®, Microsoft Deutschland GmbH, Unterschleißheim). The statistical analysis and figures were performed with SPSS (Statistical Product and Services Solutions, Version 25, SPSS Inc., Chicago, IL, USA) and Graphpad Prism (Version V, GraphPad Software, La Jolla, USAGraph Pad). Descriptive statistics were carried out for the complete dataset. The Student`s t-test and one-way Anova with Kruskal-Wallis, as well as the Dunns-Multiple Comparison and Mann-Whitney-U Test, were used for the determination of significance (*=*P* < 0.05, **=*P* < 0.01, ***=*P* < 0.001).

## Results

In total, 104 individuals divided in 38 female and 66 male patients were included in the present retrospective analysis of patients with mild FXIII deficiency (≤70%) treated at the ICU. The lowest FXIII levels measured ranged from 18.8% to 70% (Table 1).

**Table 1.** Patient Characteristics of the Analyzed Cohort.

Parameter	Median (min-max)
Age [years]	57 (18-86)
Weight [kg]	67.7 (34-130)
Lowest FXIII Level [%]	47.4 (18.8-69.8)
Lowest Prothrombin Time [%]	51.5 (7-117)
Highest aPTT [sec]	58.5 (27-180)
Lowest Fibrinogen Level [mg/dL]	177 (60-834)
Lowest FVIII-Level [%]	136.5 (62-400)
Lowest Hemoglobin Level [g/dL]	7.6 (4.6-14.9)
Highest Hemoglobin Level [g/dL]	13.7 (10.4-19.6)
Packed Red Blood Cells (U)	24 (2-122)
Fresh Frozen Plasma (U)	17 (1-60)
PPSB (IU)	4800 (600-57600)
Thrombocyte Concentrates (U)	10 (2-195)
Given Fibrinogen (g)	6 (1-68)
Antithrombin-III (IU)	6000 (500-54500)
FXIII-Substitution (IU)	2500 (1250-51250)

The patient collective was separated by different causes of stay at the ICU, differentiated between major interventions (e.g., abdominal, heart, vascular surgery; n = 39), minor interventions (e.g., gastroscopy, tonsillectomy, drains; n = 9), primary trauma (n = 12), and a spontaneous bleeding event (n = 24). There was no statistical difference in FXIII activity levels between the analyzed groups regarding the cause of ICU stay (*P* = 0.8375).

**Figure 1.**
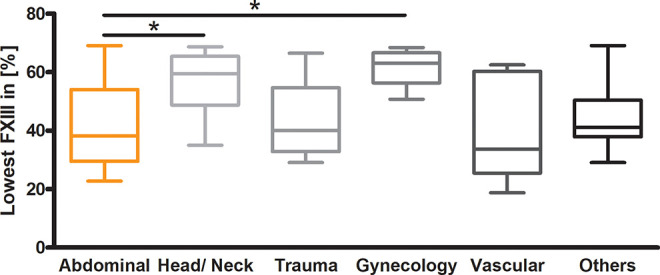
Lowest measured factor XIII activity related to focus of illness. Comparison of different illness foci: abdominal (n = 37), head/neck (n = 13), trauma (n = 12), gynecology (n = 5), vascular (n = 7), others (n = 8). The Kruskal-Wallis test was performed with *P* = 0.0027, Dunn’s Multiple Comparison test is indicated by *=*P* < 0.05.

The lowest FXIII levels were also compared by the bleeding focus in the 6 different groups: Abdominal, Head/ Neck, Trauma, Gynecology, Vascular, and Others. Visually, the results indicate higher minimum FXIII values for a Head/ Neck or Gynecology Focus. In comparison, patients with an abdominal focus in particular had significantly lower values of FXIII (Abdominal vs. Head/ Neck *P* < 0.05, Abdominal vs. Gynecology *P* < 0.05, *P* = 0.0027) (Figure 1).

**Figure 2.**
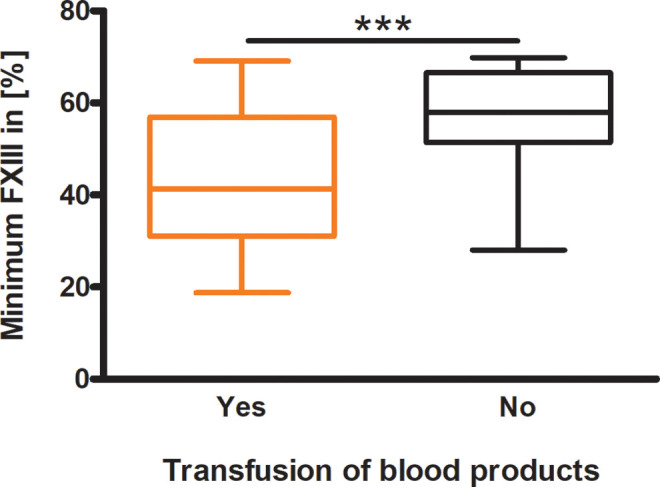
Differences in factor XIII activity divided by need of transfusion. Patients who needed a transfusion during ICU stay were compared with those who did not receive blood products at any time (n = 83 vs. n = 21); the Student’s t-test was performed with *P* = 0.0003. *P* < 0.001=***.

In Figure 2, the patient collective is shown separated by the need of any kind of blood products. The graph demonstrates that the median FXIII value was significantly lower in the patients treated with blood products compared to the group without any transfusion (41.3% vs. 57.9%; *P* < 0.001).

To evaluate the clinical relevance of a mild deficiency, different FXIII cut-off values were defined. Afterwards, the patient collective was separated by the varying FXIII cut-offs and the groups were compared with their clinical course. The cut-offs of 60% (<60 n = 78, ≥60% n = 26), 50% (<50 n = 56, ≥50% n = 48), 40% (<40 n = 39, ≥40% n = 65), and 30% (<30 n = 20, ≥30% n = 84) were analyzed.

**Figure 3.**
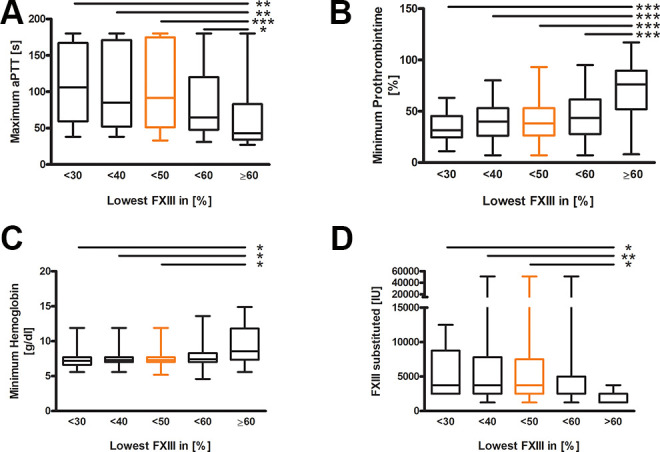
Comparison of factor XIII activity cut-offs. A and B, Comparing the different groups of minimum factor XIII activity levels shows that the standard coagulation parameters max. partial thromboplastin time (<30 n = 20, <40 n = 39, <50 n = 56, <60 n = 78, ≥60 n = 24; spearman R2 = −0.34, *P* = 0.001) and min. prothrombin time (<30 n = 20, <40 n = 39, <50 n = 56, <60 n = 78, ≥60 n = 26; Spearman R2 = 0.49, *P* < 0.001) correlate negatively and positively with the minimum FXIII activity, respectively. C, Shows the minimum hemoglobin values (<30 n = 20, <40 n = 39, <50 n = 56, <60 n = 78, ≥60 n = 26) that differ significantly beginning at FXIII<50% compared to ≥60%. D, The substitution of FXIII units is shown for the different groups of minimum FXIII activity level (<30 n = 11, <40n = 22, <50 n = 30, <60 n = 43, ≥60 n = 9). The Kruskal-Wallis test was performed; Dunn`s Multiple Comparison test is indicated by *P* < 0.05=*, *P* < 0.01=**, *P* < 0.001=***.

We also compared the different FXIII cut-offs with other standard coagulation parameters. The global coagulation parameters of maximum activated partial thromboplastin time (aPTT) (*P* = 0.0003), minimum prothrombin time (*P* = 0.0001), and minimum fibrinogen level (not shown, *P* = 0.0004) showed significant differences between patients with FXIII values above and below 60% (Figure 3A and B), indicating a global impact on coagulation systems with a hampered pro-coagulatory capacity. Interestingly, FVIII values were not different between the groups (not shown, *P* = 0.8713).

The mentioned difference in FXIII levels between patients with and without transfusion could also be shown vice-versa. Comparing the groups with FXIII levels ≥60% and the other cut-offs results in significantly lower minimum hemoglobin values beginning at <50% (*P* = 0.0125) (Figure 3C). The maximum hemoglobin level did not differ between the different analyzed cut-offs of FXIII (not shown). However, in the patients with FXIII cut-off values <50%, significantly more units of FXIII had been substituted (*P* < 0.005) (Figure 3D).

**Figure 4.**
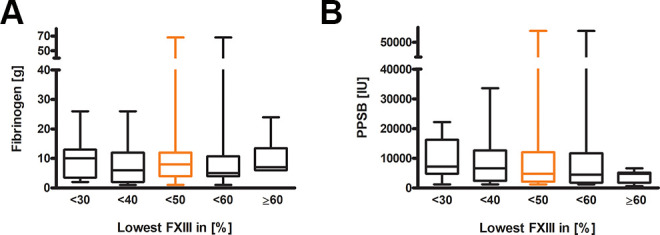
Amount of substituted clotting factors for different minimal factor XIII activity cut-offs. (A) Shows the substitution of fibrinogen (<30 n = 10, <40 n = 17, <50 n = 27, <60 n = 36, ≥60 n = 6) and (B) shows PPSB (<30 n = 11, <40 n = 19, <50 n = 29, <60 n = 34, ≥60 n = 7) compared between groups with different minimum FXIII activities and ≥60 % minimum FXIII activity. The Kruskal-Wallis test was performed; Dunn`s Multiple Comparison test is indicated by *P* < 0.05=*, *P* < 0.01=**, *P* < 0.001=***.

Despite the differences in laboratory clotting tests, the pro-coagulatory management over time was quite similar: the amounts of PPSB, fibrinogen and AT-III (not-shown) given did not differ between the groups (Figure 4A and B).

**Figure 5.**
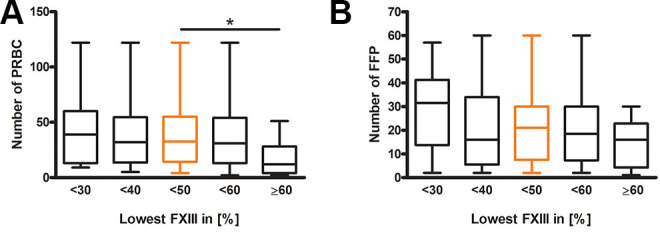
Amount of blood transfusions for different minimal factor XIII activity cut-offs. (A) Shows the substitution of packed red blood cells (PRBC) (<30 n = 19, <40 n = 37, <50 n = 52, <60 n = 65, ≥60 n = 15) and (B) fresh frozen plasma (FFP) (<30 n = 16, <40 n = 33, <50 n = 45, <60 n = 52, ≥60 n = 12) compared between groups with different minimum FXIII activities and ≥60 % minimum FXIII activity. The Kruskal-Wallis test was performed; Dunn`s Multiple Comparison test is indicated by *P* < 0.05=*, *P* < 0.01=**, *P* < 0.001=***.

This also correlates with the number of given packed red blood cells (PRBC), which was higher in patients with FXIII Levels <50% (*P* = 0.0421) (Figure 5A). On the other hand, there was no difference in the amount of FFP given to patients of the different cut-offs (*P* = 0.1916) (Figure 5B).

Table 2 shows the characteristics of the patient collective separated by a 50% cut-off for the lowest measured FXIII value. Patients with FXIII levels <50 received significantly more FXIII-products. There is a slight difference in age between the groups, but no statistically significant difference in body weight. The minimum and maximum hemoglobin values measured were significantly lower in patients with FXIII <50%; consequently, the rate of PRBC transfusions was higher in this group. Similarly, the measured coagulation parameters also differed significantly between the collectives, but management with pro-coagulatory substances, like fibrinogen, PPSB, AT-III, and FFP, showed no differences. The rate of thrombocyte transfusion also showed no group difference.

**Table 2.** Patient Characteristics Comparing Minimum FXIII Activity <50% and ≥50%.^a^

	Lowest factor XIII level <50%, mean (min-max)	Lowest factor XIII level ≥50%, mean (min-max)	Significance level^b^
Age [years]	59 (19-86)	50 (18-79)	**0.0103**
Weight [kg]	70.6 (34-130)	74.1 (52-113)	0.5563
Lowest FXIII Level [%]	35 (18.8-49.6)	61 (50-70)	**<0.0001**
Lowest Prothrombin Time [%]	40 (7-93)	66 (8-117)	**<0.0001**
Highest aPTT [sec]	104 (33-180)	63 (27-180)	**<0.0001**
Lowest Fibrinogen Level [mg/dL]	163.0 (60-600)	227.8 (60-837)	**0.0005**
Lowest FVIII-Level [%]	177 (62-400)	176 (81-395)	0.6635
Lowest Hemoglobin Level [g/dL]	7.6 (5.2-11.9)	9.1 (4.6-14.9)	**0.0044**
Highest Hemoglobin Level [g/dL]	13.4 (10.4-17.4)	14.3 (11.3-17.1)	**0.0046**
Packed Red Blood Cells (U)	40.48 (4-122)	21 (2-64)	**0.0017**
Fresh Frozen Plasma (U)	21 (2-60)	17 (1-60)	0.2278
PPSB (IU)	9282 (1200-57600)	6750 (600-44400)	0.2041
Thrombocytes Concentrates (U)	19 (2-195)	11 (2-36)	0.1280
Given Fibrinogen (g)	10.5 (1-68)	8.5 (2-39)	0.3408
Antithrombin-III-Subst. (IU)	11286 (500-54500)	9208 (2000-35500)	0.7309
FXIII-Substitution (IU)	6417 (1250-51250)	2102 (1250-3750)	**0.0002**

^a^ Statistical analysis was performed with the Mann-Whitney-U-test.

^b^Boldface values correspond to significant results with *P* < 0.05.

## Discussion

Clinical data suggest that mild FXIII deficiency is an underdiagnosed and underrepresented disease entity that is common in the perioperative setting. The analysis of Lawrie et al, with a sample of >1000 patients, showed that 21% of the hospitalized patients had a FXIII activity out of the normal range (<70%), and 6% reached values <50%.^29^ Some publications suggest that there is no correlation between FXIII activity and bleeding events or transfusion of blood products.^30,31^ However, Rappard et al demonstrated that FXIII was the most important independent variable of clot firmness, and in different studies the preoperative treatment with FXIII (FXIII activity target >70%) significantly reduced the need of transfusion as well as the perioperative blood loss.^32-34^

The results of the present analysis of 104 patients with mild FXIII deficiency strengthen the clinical relevance of FXIII in the intensive care setting and support the association between FXIII activity and transfusion of blood products (Figure 2). Comparing the different groups of patients, those with lower FXIII levels, beginning at <60%, showed lower minimum and maximum hemoglobin values (Figure 3C). Furthermore, our data support the results of Song et al that a reduced FXIII activity correlates significantly with general coagulation markers, such as prothrombin time, aPTT, and fibrinogen which show reduced values for lower FXIII levels (Figure 3A and B).^35^ These comparisons match with the finding, that in the underlying analysis the patients with FXIII activities <50% had a significantly higher need for PRBC Transfusion (Figure 5), even though the amount of given FFP, thrombocytes, PPSB, AT-III, and fibrinogen did not differ (Table 2).

To classify these results the (patho-)physiology of FXIII plays an important role. The symptoms of mild FXIII deficiency consist of delayed bleeding events, occurring 12-36 h after a trauma, probably caused by a reduced clot firmness, which correlates with FXIII activity and early fibrinolysis by a lack of alpha-2 plasmin inhibitor crosslinking.^10,11,15^ A previous study actually indicates that cross linking requires FXIII activity levels of at least 30%, but is completed only at much higher levels.^36^ This could be an indicator that mild FXIII deficiency, as analyzed in the present study, particularly plays a role in disseminated minor bleeding events, which become clinically relevant after a longer period of bleeding. This hypothesis correlates with the results of another study, showing that patients with mild FXIII deficiency had higher drainage volumes postoperatively.^37^

Comparing the amount of FXIII substitution in the present study between the groups of FXIII activities below and over 50%, patients with lower FXIII levels were consequently substituted with 3 times more units of FXIII (Figure 3D). Since further there has been no difference in the need for pro-coagulatory treatment between the 2 groups, the higher rate of PRBC transfusion in the patients with FXIII <50% must be explained otherwise. Taking into consideration, that the half-life of FXIII is about 10-12 days, this could be a sign of a higher FXIII consumption, e.g. in the case of prolonged bleeding or as part of disseminated intravasal coagulopathy, an effect that has already been described.^35,38,39^

The results of the underlying analysis further suggest that the cause of bleeding, compared between spontaneous events, trauma, and major or minor surgery, has no influence on the lowest measured FXIII activity. More important seems to be the focus of illness and/or bleeding event. Patients with an abdominal focus showed significantly lower FXIII activities than head/neck or gynecological foci (Figure 1).

Limitations of this study are the retrospective design, which is only able to generate new hypotheses, and the fact that there were just minimum and maximum values of FXIII activities, but no repeated measurements for the complete period of ICU stay. Nevertheless, a minimum FXIII activity below 50% measured at any point in the course of the disease was associated with a significantly higher need for PRBC transfusion. In the patient groups with FXIII levels <40% and <30% there was only a non-significant trend toward a higher need for PRBC transfusion, probably because the analysis was under-powered due to the reduced number of patients in these groups (Figure 5).

Further prospective randomized studies are needed to confirm these results and strengthen the evidence regarding the clinical relevance of mild acquired FXIII deficiency.

## Conclusion

Taken together, these results indicate that a mild FXIII deficiency occurring at any point of ICU stay is also probably relevant for the total need of PRBC, independent of pro-coagulatory management, which did not differ between the groups. Based on the underlying data and the literature, it is important raising awareness of mild FXIII deficiency in the intensive care setting and we recommend providing routine measurements in patients at high risk of bleeding. At present, in alignment with the ESAIC guidelines, an early management with the substitution of FXIII at levels <50-60% could be suggested.^32,40^

## Abbreviations

aPTT: activated partial thromboplastin time
FXIII: Factor XIII
FFP: fresh frozen plasma
PRBC: packed red blood cells.

## References

[1] Schroeder V Kohler HP . New developments in the area of factor XIII. J Thromb Haemost. 2013;11(2):234–244. doi:10.1111/jth.12074 23279671 10.1111/jth.12074

[bibr2-10760296211024741] Muszbek L Bereczky Z Bagoly Z Komáromi I Katona É. Factor XIII: a coagulation factor with multiple plasmatic and cellular functions. Physiol Rev. 2011;91(3):931–972. doi:10.1152/physrev.00016.2010 21742792 10.1152/physrev.00016.2010

[bibr3-10760296211024741] Hellerer O Brückner WL Frey KW Westerburg KW Klessinge U . Fracture healing under factor XIII medication. Arch Orthop Trauma Surg. 1980;97(2):157–159. doi:10.1007/BF00450939 7458602 10.1007/BF00450939

[4] Duckert F Jung E Shmerling DH . A hitherto undescribed congenital haemorrhagic diathesis probably due to fibrin stabilizing factor deficiency. Thromb Diath Haemorrh. 1960;5:179–186. http://www.ncbi.nlm.nih.gov/pubmed/13724728 13724728

[5] Schwartz ML Pizzo S V Hill RL McKee PA . Human factor XIII from plasma and platelets. Molecular weights, subunit structures, proteolytic activation, and cross-linking of fibrinogen and fibrin. J Biol Chem. 1973;248(4):1395–1407. doi:10.1016/S0021-9258(19)44312-3 4405643

[bibr6-10760296211024741] Ádány R Kiss A Muszbek L . Factor XIII: a marker of mono-and megakaryocytopoiesis. Br J Haematol. 1987;67(2):167–172. doi:10.1111/j.1365-2141.1987.tb02321.x 3314976 10.1111/j.1365-2141.1987.tb02321.x

[7] Mitchell JL Lionikiene AS Fraser SR Whyte CS Booth NA Mutch NJ . Functional factor XIII-A is exposed on the stimulated platelet surface. Blood. 2014;124(26):3982–3990. doi:10.1182/blood-2014-06-583070 25331118 10.1182/blood-2014-06-583070 PMC4309449

[8] Bolton-Maggs PHB Perry DJ Chalmers EA , et al. The rare coagulation disorders—review with guidelines for management from the United Kingdom Haemophilia Centre Doctors’ Organisation. Haemophilia. 2004;10(5):593–628. doi:10.1111/j.1365-2516.2004.00944.x 15357789 10.1111/j.1365-2516.2004.00944.x

[9] Anwar R Gallivan L Edmonds SD Markham AF . Genotype/phenotype correlations for coagulation factor XIII: specific normal polymorphisms are associated with high or low factor XIII specific activity. Blood. 1999;93(3):897–905. doi:10.1182/blood.v93.3.897 9920838

[10] McKee PA Schwartz ML Pizzo SV Hill RL . Cross-linking of fibrin by fibrin-stabilizing factor. Ann N Y Acad Sci. 1972;202:127–148. doi:10.1111/j.1749-6632.1972.tb16326.x 4565368 10.1111/j.1749-6632.1972.tb16326.x

[11] Sakata Y Aoki N . Cross-linking of α2-plasmin inhibitor to fibrin by fibrin-stabilizing factor. J Clin Invest. 1980;65(2):290–297. doi:10.1172/JCI109671 6444305 10.1172/JCI109671 PMC371366

[12] Peyvandi F Mannucci PM . Rare coagulation disorders. Thromb Haemost. 1999;82(4):1207–1214. http://www.ncbi.nlm.nih.gov/pubmed/10544899 10544899

[13] Biswas A Ivaskevicius V Thomas A Oldenburg J . Coagulation factor XIII deficiency. Diagnosis, prevalence and management of inherited and acquired forms. Hamostaseologie. 2014;34(02):160–166. doi:10.5482/HAMO-13-08-0046 24503678 10.5482/HAMO-13-08-0046

[14] Ichinose A . Factor XIII is a key molecule at the intersection of coagulation and fibrinolysis as well as inflammation and infection control. Int J Hematol. 2012;95(4):362–370. doi:10.1007/s12185-012-1064-3 22477542 10.1007/s12185-012-1064-3

[15] Egbring R Kröniger A Seitz R. Factor XIII deficiency: pathogenic mechanisms and clinical significance. Semin Thromb Hemost. 1996;22(5):419–425. doi:10.1055/s-2007-999041 8989826 10.1055/s-2007-999041

[16] Anwar R Minford A Gallivan L Trinh CH Markham AF . Delayed umbilical bleeding—a presenting feature for factor XIII deficiency: clinical features, genetics, and management. Pediatrics. 2002;109(2): E32. doi:10.1542/peds.109.2.e32 11826242 10.1542/peds.109.2.e32

[17] Adam EH Meier J Klee B , et al. Factor XIII activity in patients requiring surgical re-exploration for bleeding after elective cardiac surgery—a prospective case control study. J Crit Care. 2020;56:18–25. doi:10.1016/j.jcrc.2019.11.012 31805464 10.1016/j.jcrc.2019.11.012

[18] Saeki H Masuda T Okada S , et al. Impact of perioperative peripheral blood values on postoperative complications after esophageal surgery. Surg Today. 2010;40(7):626–631. doi:10.1007/s00595-009-4135 -1 20582513 10.1007/s00595-009-4135-1

[19] Lim W Moffat K Hayward CPM . Prophylactic and perioperative replacement therapy for acquired factor XIII deficiency. J Thromb Haemost. 2004;2(6):1017–1019. doi:10.1111/j.1538-7836.2004.00728.x 15140148 10.1111/j.1538-7836.2004.00728.x

[bibr20-10760296211024741] Janbain M Nugent DJ Powell JS St-Louis J Frame VB Leissinger CA . Use of factor XIII (FXIII) concentrate in patients with congenital FXIII deficiency undergoing surgical procedures. Transfusion. 2015;55(1):45–50. doi:10.1111/trf.12784 25070582 10.1111/trf.12784

[bibr21-10760296211024741] Korte W. F XIII in perioperative coagulation management. Best Pract Res Clin Anaesthesiol. 2010;24(1):85–93. doi:10.1016/j.bpa.2009.09.011 20402172 10.1016/j.bpa.2009.09.011

[22] Gerlach R Tölle F Raabe A Zimmermann M Siegemund A Seifert V. Increased risk for postoperative hemorrhage after intracranial surgery in patients with decreased factor XIII activity: implications of a prospective study. Stroke. 2002;33(6):1618–1623. doi:10.1161/01.STR.0000017219.83330.FF 12053001 10.1161/01.str.0000017219.83330.ff

[23] Nugent D. Corifact^TM^/Fibrogammin® P in the prophylactic treatment of hereditary factor XIII deficiency: results of a prospective, multicenter, open-label study. Thromb Res. 2012;130(suppl 2):S12–S14. doi:10.1016/S0049-3848(13)70005-7 10.1016/S0049-3848(13)70005-7 23439001

[24] Muszbek L Katona É . Diagnosis and management of congenital and acquired FXIII deficiencies. Semin Thromb Hemost. 2016;42(4):429–439. doi:10.1055/s-0036-1572326 27071048 10.1055/s-0036-1572326

[25] Solomon C Korte W Fries D , et al. Safety of factor XIII concentrate: analysis of more than 20 years of pharmacovigilance data. Transfus Med Hemother. 2016;43(5):365–373. doi:10.1159/000446813 27781024 10.1159/000446813 PMC5073543

[26] Inbal A Oldenburg J Carcao M Rosholm A Tehranchi R Nugent D. Recombinant factor XIII: a safe and novel treatment for congenital factor XIII deficiency. Blood. 2012;119(22):5111–5117. doi:10.1182/blood-2011-10-386045 22451421 10.1182/blood-2011-10-386045

[27] Ivaskevicius V Biswas A Bevans C , et al. Identification of eight novel coagulation factor XIII subunit a mutations: implied consequences for structure and function. Haematologica. 2010;95(6):956–962. doi:10.3324/haematol.2009.017210 20179087 10.3324/haematol.2009.017210 PMC2878794

[28] Mangla A Hamad H Kumar A . Factor XIII deficiency. Published 2021. Accessed May 3, 2021. http://www.ncbi.nlm.nih.gov/pubmed/32491399 32491399

[29] Lawrie AS Green L Mackie IJ Liesner R Machin SJ Peyvandi F . Factor XIII—an under diagnosed deficiency—are we using the right assays? J Thromb Haemost. 2010;8(11):2478–2482. doi:10.1111/j.1538-7836.2010.04028.x 20727071 10.1111/j.1538-7836.2010.04028.x

[30] Fahlbusch FB Heinlein T Rauh M , et al. Influence of factor XIII activity on post-operative transfusion in congenital cardiac surgery—a retrospective analysis. In: Erdoes G , ed. PLoS One. 2018;13(7): e0199240. doi:10.1371/journal.pone.0199240 29990321 10.1371/journal.pone.0199240 PMC6038983

[31] Adelmann D Klaus DA Illievich UM , et al. Fibrinogen but not factor XIII deficiency is associated with bleeding after craniotomy. Br J Anaesth. 2014;113(4):628–633. doi:10.1093/bja/aeu133 24871873 10.1093/bja/aeu133

[32] Dickneite G Herwald H Korte W Allanore Y Denton CP Cerinic MM . Coagulation factor XIII: a multifunctional transglutaminase with clinical potential in a range of conditions. Thromb Haemost. 2015;113(4):686–697. doi:10.1160/TH14-07-0625 25652913 10.1160/TH14-07-0625

[bibr33-10760296211024741] Korte WC Szadkowski C Gähler A , et al. Factor XIII substitution in surgical cancer patients at high risk for intraoperative bleeding. Anesthesiology. 2009;110(2):239–245. doi:10.1097/ALN.0b013e318194b21e 19194150 10.1097/ALN.0b013e318194b21e

[34] von Rappard S Hinnen C Lussmann R Rechsteiner M Korte W . Factor XIII deficiency and thrombocytopenia are frequent modulators of postoperative clot firmness in a surgical intensive care unit. Transfus Med Hemother. 2017;44(2):85–92. doi:10.1159/000468946 28503124 10.1159/000468946 PMC5425767

[35] Song JW Choi JR Song KS Rhee J-H . Plasma factor XIII activity in patients with disseminated intravascular coagulation. Yonsei Med J. 2006;47(2):196–200. doi:10.3349/ymj.2006.47.2.196 16642548 10.3349/ymj.2006.47.2.196 PMC2687628

[36] Francis CW Marder VJ . Rapid formation of large molecular weight α-polymers in cross-linked fibrin induced by high factor XIII concentrations: role of platelet factor XIII. J Clin Invest. 1987;80(5):1459–1465. doi:10.1172/JCI113226 3680507 10.1172/JCI113226 PMC442404

[37] Gödje O Haushofer M Lamm P Reichart B . The effect of factor XIII on bleeding in coronary surgery. Thorac Cardiovasc Surg. 1998;46(5):263–267. doi:10.1055/s-2007-1010236 9885116 10.1055/s-2007-1010236

[38] Miloszewski K Losowsky MS . The half-life of factor XIII in vivo. Br J Haematol. 1970;19(6):685–690. doi:10.1111/j.1365-2141.1970.tb07013.x 5492488 10.1111/j.1365-2141.1970.tb07013.x

[39] Johansson PI Sørensen A Perner A , et al. Disseminated intravascular coagulation or acute coagulopathy of trauma shock early after trauma? An observational study. Crit Care. 2011; 15(6):R272. doi:10.1186/cc10553 22087841 10.1186/cc10553 PMC3388658

[40] Kozek-Langenecker SA Afshari A Albaladejo P , et al. Management of severe perioperative bleeding: guidelines from the European Society of Anaesthesiology. Eur J Anaesthesiol. 2013;30(6):270–382. doi:10.1097/EJA.0b013e32835f4d5b 23656742 10.1097/EJA.0b013e32835f4d5b

